# Development and Validation of a Functional Behavioural Assessment Ontology to Support Behavioural Health Interventions

**DOI:** 10.2196/medinform.7799

**Published:** 2018-05-31

**Authors:** Gianluca Merlo, Giuseppe Chiazzese, Davide Taibi, Antonella Chifari

**Affiliations:** ^1^ Istituto per le Tecnologie Didattiche Consiglio Nazionale delle Ricerche Palermo Italy

**Keywords:** ontology, behavioral interventions, functional behavioral assessment, eHealth care, evidence-based practice

## Abstract

**Background:**

In the cognitive-behavioral approach, Functional Behavioural Assessment is one of the most effective methods to identify the variables that determine a problem behavior. In this context, the use of modern technologies can encourage the collection and sharing of behavioral patterns, effective intervention strategies, and statistical evidence about antecedents and consequences of clusters of problem behaviors, encouraging the designing of function-based interventions.

**Objective:**

The paper describes the development and validation process used to design a specific Functional Behavioural Assessment Ontology (FBA-Ontology). The FBA-Ontology is a semantic representation of the variables that intervene in a behavioral observation process, facilitating the systematic collection of behavioral data, the consequential planning of treatment strategies and, indirectly, the scientific advancement in this field of study.

**Methods:**

The ontology has been developed deducing concepts and relationships of the ontology from a gold standard and then performing a machine-based validation and a human-based assessment to validate the Functional Behavioural Assessment Ontology. These validation and verification processes were aimed to verify how much the ontology is conceptually well founded and semantically and syntactically correct.

**Results:**

The Pellet reasoner checked the logical consistency and the integrity of classes and properties defined in the ontology, not detecting any violation of constraints in the ontology definition. To assess whether the ontology definition is coherent with the knowledge domain, human evaluation of the ontology was performed asking 84 people to fill in a questionnaire composed by 13 questions assessing concepts, relations between concepts, and concepts’ attributes. The response rate for the survey was 29/84 (34.52%). The domain experts confirmed that the concepts, the attributes, and the relationships between concepts defined in the FBA-Ontology are valid and well represent the Functional Behavioural Assessment process.

**Conclusions:**

The new ontology developed could be a useful tool to design new evidence-based systems in the Behavioral Interventions practices, encouraging the link with other Linked Open Data datasets and repositories to provide users with new models of eHealth focused on the management of problem behaviors. Therefore, new research is needed to develop and implement innovative strategies to improve the poor reproducibility and translatability of basic research findings in the field of behavioral assessment.

## Introduction

### Background

Behavioral Interventions (BI) are assessed as effective and evidence-based strategies by several studies and meta-analyses for reducing problem behaviors identified in school-age children from [1-5]. Among BI the Functional Behavioral Assessment (FBA) is considered one of the most effective methods for identifying the antecedents and consequences that control a problem behavior [6] and for gathering information about the reason or function for a behavior [7]. In the FBA, the data obtained through indirect measures, direct observation, and experimental manipulation of environmental variables contribute in formulating a functional hypothesis.

It can then be used in designing effective intervention plans aimed at reducing the reinforcement effect that specific antecedents and consequents could have in triggering and maintaining the problem behavior. For instance, children with Attention Deficit Hyperactivity Disorder (ADHD), one of the most common syndromes generating behavior disorders, often show many disruptive behaviors during class at school. If appropriate instructional methodologies are not implemented by teachers, a child with ADHD can have difficulties in sustaining attention to a task, and this can trigger challenging classroom behavior.

For example, these include: calling out, leaving their seat, and frequent rule violations. If the FBA was applied in a similar case, health professionals would probably have hypothesized that the function “avoidance” is what motivates the child´s behavior in an attempt to get away from the frustrating task. Accordingly, they would have suggested teachers use an intervention plan composed of strategies aimed at increasing the student task-oriented behaviors. These include the following: breaking the task into smaller portions, reducing the task duration, using visual cues, and reducing the number of challenging ones.

Newcomer and Lewis [8], comparing treatment outcomes demonstrate that behavior intervention plans based on FBA information (function-based) were more effective than behavior intervention plans not based on FBA information (non-function-based). This confirms the usefulness and importance of conducting an FBA to guide intervention plans based on the conscientious and explicit use of current best evidence [9].

The general tendency of the scientific community to open and share processes and results to anyone interested could be a further opportunity to corroborate the application of FBA as Evidence-Based Practice (EBP). An EBP is a decision-making process that integrates: the best available evidence, clinical expertise, client values, and context [10]. As suggested by Kazdin [11], clinical psychology “would profit enormously from codifying the experiences of the clinician in practice so that the information is accumulated and can be drawn on to generate and test hypotheses”. Transparency, openness, and, reproducibility could be the lifeblood for the advancement of psychological sciences and the dissemination of a more open research culture [12]. However, Scott and Alter [13], reveal that only a few scientific papers about FBA with an EBP approach can be found in the literature.

In this direction, Richesson and Andrews [14] explore how computer science could support the digitization and computation of information related to clinical processes regarding representation of knowledge found in clinical studies and in particular the role of ontologies.

In computer science, an ontology is a taxonomic description of the concepts in an application domain and the relationships among them [15] aimed to promote knowledge generation, organization, reuse, integration, and analysis [16]. Ontologies are a powerful tool to accumulate knowledge in a specific domain especially when there is a lack of shared terms and procedures.

Today, the use of ontologies in biomedical research is an established practice. For example, Gene Ontology [17] provides researchers with extensive knowledge regarding the functions of genes and gene products. Also, the Open Biomedical Ontologies initiative provides a repository of controlled vocabularies to be used across different biological and medical domains [18]. However, computable information about behavioral disorders and mental illness is still dispersed. The lack of shared definition and practices makes them difficult to aggregate, share, and search for specific information when needed.

Recently, researchers have started to recognize the important role that ontologies can play in the clinical psychology context. By far the most interesting examples include the: (1) Mental Disease Ontology [19], (2) Mental Health Ontology [20], (3) Mood Disorder Ontology [21], (4) Autism Phenotype Ontology [22], (5) Ontology of Schizophrenia [23], and (6) Ontology to monitor mental retardation rehabilitation process [24].

In the domain of the description of human behavior, a successful example is the Ontology for human behavior models [25] created with the purpose of tracing what causes a person to take an action, the cognitive state associated with the behavior, and the effects of the particular action. However, at the time of writing this paper, authors have not identified ontologies specifically focused on behavioral disorders according to the FBA methods in the main international journals on medical information systems. Starting from this perspective, the definition of a Functional Behavior Ontology (FBA-Ontology) could play a key role in the adoption of an evidence-based approach among behavioral experts to fill the gap between research and practice still widely observed in clinical psychology [26]. In fact, data mining algorithms have great potential for identifying patterns in psychological data, facilitating the decision-making processes, and automatic meta-analysis.

This study presents the description and validation of the FBA-Ontology [27] as a semantic tool to support the systematic collection of behavioral knowledge and the decision-making process based on evidence and gathered data.

## Methods

### Methodological Approach

The FBA-Ontology was developed applying the Uschold and King [28] methodology, which comprises the following set of guidelines: (1) identification of the ontology purpose, (2) capture the concepts and the relations between the concepts, (3) coding the ontology using a formal language, and (4) evaluate the ontology from a technical point of view. Moreover, authors of the present contribution also added a human-based assessment by interviewing 84 domain experts to check the formal structure of the ontology regarding taxonomy, relationships, and axioms. The next paragraphs describe in detail each of the steps as mentioned above.

### Identification of the Ontology Purpose

The FBA-Ontology purpose is to describe the structure and the semantics of Functional Behavior Assessment methods. Gresham et al [29] define the FBA as “a collection of methods for gathering information about antecedents, behaviors, and consequences to determine the reason (function) of behavior.” The FBA derives from operant learning theories [30,31], and it is commonly used in clinical and educational contexts to design effective intervention plans. These are aimed at reducing the reinforcement effect that specific antecedents and consequents could have in triggering and maintaining the problem behavior.

### Capture the Concepts and the Relations Between the Concepts

**Figure 1:**
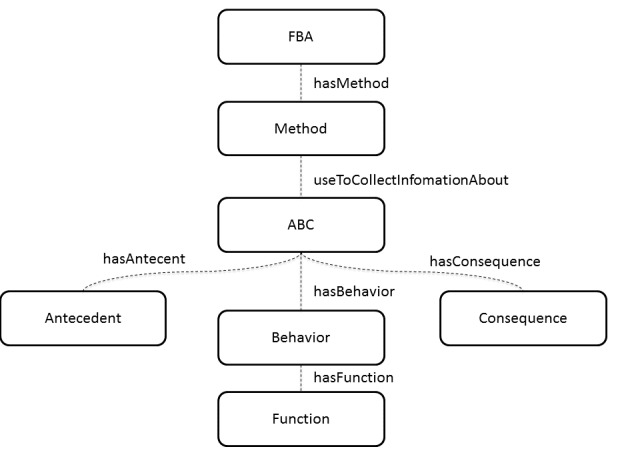
Key concepts at the basis of the FBA-Ontology.

The concepts of the ontology were captured starting from the above mentioned theoretical assumptions. The FBA-Ontology is, therefore, a collection of classes and properties used to describe the whole assessment process. This includes the definition of a target behavior, the collection of the behavioral data, the hypotheses about the target behavior functions, and the planning of a behavioral intervention. In particular, the FBA-Ontology key concepts are FBA, Method, Antecedent, Behavior, Consequence, and Function (Figure 1).

According to Hanley [32], the FBA is a descriptive assessment including indirect and direct observation methods and measurements of a target behavior. The FBA-Ontology includes the *Method* class to specify the observation methods applied to the target behavior, specified in the class *Behavior*. Rating scales, questionnaires, and interviews are examples of indirect methods because they do not require direct observation of the target behavior. The direct methods are based on descriptive assessments and systematic recordings of observation sessions. The descriptive assessments provide qualitative information about variables that may trigger or maintain a target behavior, while the recording methods, such as the systematic direct observation, provide quantitative information about frequency, intensity, and duration of a targeted behavior during a specific time interval. The property *isDirect*, defined within the *Method* class, models the use of several direct or indirect methods [33].

The triad of classes: *Antecedent*, *Behavior*, and *Consequence* encloses the descriptors of the target behavior (Behavior), and the variables that trigger (Antecedent) or maintain it (Consequence). The class *Function* defines what purpose the problem behavior serves for the individual. According to Iwata and colleagues [34], the four behavioral functions are: avoid or escape difficult tasks, gain adult and peer attention, access to a desired object or activity, and sensory stimulation. The FBA-Ontology embodies these functions through the purported enumerated *datatypes*, included into the *Function* class mentioned above.

**Figure 2:**
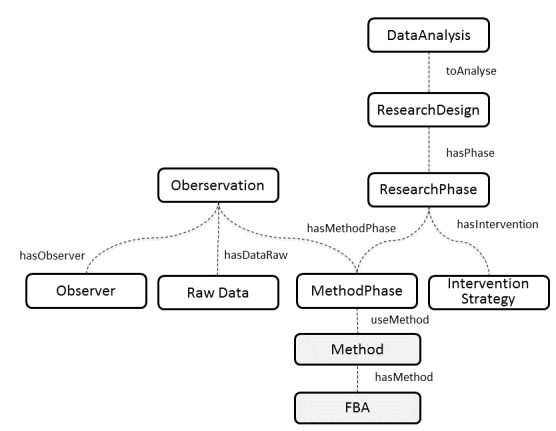
Key concepts related to research methods and data collection.

Unlike experimental designs where researchers can randomly assign participants to a control and treatment group, the behavior of the subject under observation generates data that can change over time or stay steady. To evaluate whether the time series changes, the elective and most popular research approach is single case research design. Single-case research designs are a diverse and powerful set of procedures used for demonstrating causal relationships among clinical phenomena [35]. Clinicians use three main research designs: case studies, quasi-experimental designs, and experimental designs. The differences among these are regarded as the increasing level of scientific rigor, ranging from anecdotal data gathered retrospectively to the maximum level of control of the dependent variables achievable in a laboratory setting. Dallery and Raiff [36] suggest the use of single-case design as a method for optimizing behavioral health interventions and facilitating the practitioners in the planning of suitable interventions for both individuals and groups. The FBA-Ontology assumes that data about an observed behavior is collected according to the single-case research design constraints (Figure 2).

Generally, single-case designs start with a baseline phase (A) to observe the dependent variable as it appears. Once the baseline is established, the observer continues while implementing the intervention (B) to compare the time series looking for significant changes. The design just described is named AB. Other examples of single-case designs are ABA (adding a nontreatment condition to the AB design), ABAB (repeating the AB design twice) or BAB (implementing the intervention immediately for the safety of the person observed). The typology of single case designs used during the FBA process can be specified in the property *ResearchType* of the *ResearchDesign* class. In turn, the class *ResearchPhase* identifies the specific phase of the research design.

The *Observation* class describes the observed data gathered during a research phase. This class is linked both to the type *Observer* (the person who carries out the observation) and *RawData* (the data collected during the observation session). The class *InterventionStrategy* defines the set of strategies chosen to increase positive behaviors or decrease negative ones.

The *RawData*, once gathered during the single-case design, could be analyzed to assess the statistical effect of the intervention implemented. Many statistical methods include non-parametric tests and time-series analysis which are used to compare the data gathered during the different experimental conditions. The *DataAnalysis* class of the FBA-Ontology specifies what statistical methods are used to analyze data.

To identify the constraints related to the concepts included in the FBA-Ontology, a set of competency questions were formulated (Table 1). Competency questions are requirements that are expressed in the form of questions [37-39] using the natural language. They play an important role in both the ontology creation and validation. The competence questions support the ontology development enabling developers to identify the main elements and relationships within the selected domain. They also represent a starting point to carry out a deeper evaluation in a later stage of development [40].

### Coding the Ontology Using a Formal Language

The Protégé tool was used to model the ontology and produce an OWL (Ontology Web Language) version of the ontology. The Protégé tool also includes “reasoners” that can be used to perform inferences and to verify the ontology.

The process of ontology verification is generally performed to check its syntactic quality and the presence of anomalies or pitfalls.

In a metric proposed by Burton-Jones and colleagues [41], the syntactic quality is measured by assessing whether the source code is correctly structured, and how rich the programming language features are which model the ontology.

The anomalies or pitfalls can refer to the assessment of the logic consistency of the ontology and the identification of modeling issues in comparison with well-known best practices [42]. Many automatic tools have been developed to facilitate the ontology verification. For instance, XD Analyzer checks whether the ontology satisfies a set of best practice criteria or not, showing errors, warnings, and suggestions useful to improve it. XD Analyzer is included in XD Tools [43], and it is released as a plugin for Eclipse. Another useful recent tool is OOPS! [42] a Web-based tool aimed at identifying the most common anomalies in the ontology development. It scans 21 pitfalls grouped in 4 different dimensions: human understanding, logical consistency, real word representation, and modeling issues. Many other tools are available, but their description is out of the scope of the present paper. The FBA-ontology created with Protégé was verified by using the Pellet reasoner [44] to check the logical consistency of the ontology in addition to the integrity of classes and properties defined. The Pellet reasoner has not detected any violation of constraints in the ontology definition in its results.

### The Evaluation of the Ontology

The evaluation of the quality of an ontology plays a key role during the whole ontology development process. As suggested by Gomez-Perez [45], evaluating an ontology should ensure that it correctly implements the expected requirements and performs correctly in the real world. Low-quality ontologies reduce the possibility that intelligent agents can perform accurately intelligent tasks because of inaccurate, incomplete or inconsistent information [42]. The quality of an ontology can be assessed evaluating how well a semantic structure represents the knowledge about a specific domain and the relationships about the identified concepts. Sabou and Fernandez [46] use the term “ontology validation” to compare the ontology definitions with a frame of reference that the ontology would represent.

**Table 1:** List of competence questions formulated for the FBA-Ontology and its relative constraints.

Competency questions	Constraints
Which are the types of methods to collect information about a behavior?	Direct Indirect
How many methods can have an FBA^a^?	Unlimited number
How many functions serve a behavior?	At least 1
Which are the functions of a behavior?	Avoid or escape difficult tasks, gain adult and/or peer attention, acess to a desired object or activity, or sensory stimulation
How many antecedents for a behavior?	At least 1
How many consequences for a behavior	At least 1
How many single case research designs exist?	Many, for example: AB, ABA, ABAB^b^, multiple baseline, changing criterion
How many intervention strategies can be applied to reduce the occurrence of a behavior?	Unlimited number. (Examples: token economy, response cost, shape, etc.)
How many observers can a behavior have?	Unlimited number
Who gathers data about a behavior?	Only individuals of the class Observer
How many statistical methods can be applied to analyze the raw data?	Unlimited number

^a^FBA: Functional Behavioral Assessment.

^b^AB is a design with a baseline phase with repeated measurements. ABA and ABAB are withdrawal designs. The intervention is concluded or stopped for some period of time before it is begun again.

According to these researchers, while the ontology validation is a process to evaluate how much an ontology is well-founded and corresponds accurately to the real world, the “ontology verification” aims to evaluate whether the way in which it is produced is correct.

A wide range of approaches and methodologies can be applied to perform the ontology validation. Brank et al [47] grouped the most common methods in four categories: (1) methods comparing the ontology with a golden standard [48-53], (2) methods based on the inductive evaluation of the results obtained through the application of the ontology [54-56], (3) methods comparing the ontology with resources specialized in the ontology domain [57,58], and (4) methods based on the assessment provided by expert humans [59-62].

The evaluation strategy adopted for the FBA-ontology was based on human evaluation, and it was aimed to assess whether the ontology definition is coherent with the knowledge domain. In this case, domain experts have been interviewed to check the formal structure of the ontology regarding taxonomy, relationships, and axioms.

To let the experts in the FBA domain assess the ontology authors created a questionnaire composed of 13 questions. Questions were aimed to evaluate the issues of the ontology. In the case of concepts, experts have to rate how much they agree with a set of 12 definitions using a 5-point Likert scale (from strongly agree to strongly disagree). For relations between concepts, experts have to rate the appropriateness of 6 statements describing the links between the main concepts of the ontology using a 5-point Likert scale (from strongly agree to strongly disagree). In concepts’ attributes experts have to rate how strong the relationship between 6 concepts and 9 related attributes is through a 5-point Likert scale (from strongly related to unrelated).

Moreover, questions about demographics were included in the questionnaire to gather information about the sex, age, and level of expertise of the respondents. The following tables report concepts and attributes (Table 2) and relationships (Table 3) evaluated by the questionnaire items.

**Table 2:** Concepts and attributes of the FBA-Ontology assessed during the human-based evaluation.

Concepts	Attributes
FBA^a^	description
Behavior	description setting (ie, school, home, etc.) place
Function of a behavior	function_categories is_main_function
Typologies of behavior’s functions	category (ie, social attention, avoidance, etc)
Methods to gather information about behaviors	hasDescription isDirect
Behavioral intervention	type (reactive or proactive)
Intervention strategy	interventionType
Antecedent	description
Consequence	description
Observer	role
Research design	type (ie, AB, ABA, ABAB, etc)^b^
Data analysis	statistical method results
ResearchPhase	sequence_num

^a^FAB: Functional Behavioral Assessment.

^b^AB: is a design with a baseline phase with repeated measurements. ABA and ABAB are withdrawal designs. The intervention is concluded or stopped for some period of time before it is begun again.

**Table 3:** Relationships between concepts of the FBA-Ontology assessed during the human-based evaluation.

Relation Name	Concepts in relation
hasPhase	Research Design-Research Phases
hasFunction	Behavior-Function
has Antecedent	Behavior-Antecedent
hasConsequence	Behavior-Consequence
hasObserver	Observer-Observation
toAnalise	DataAnalysis-ResearchDesign
hasRawData	Observation-RawData
hasIntervention	Research Phase-InterventionStrategy
hasMethod	FBA^a^-Method
useToCollectInformationAbout	Method-ABC^b^

^a^FAB: Functional Behavioral Assessment.

^b^ABC is a chart to collect information about a behavior that occur in a context.

## Results

### Principal Findings

A total of 29/84 (34.52%) people accessed the survey and completed the responses. The mean age of the valid subset was 32 (SD 6.34) years with a range of 24-57 years. The respondents were mainly female (89.66%). Participants worked in the FBA domain with a mean of 5 (SD 6.87) years.

**Figure 3:**
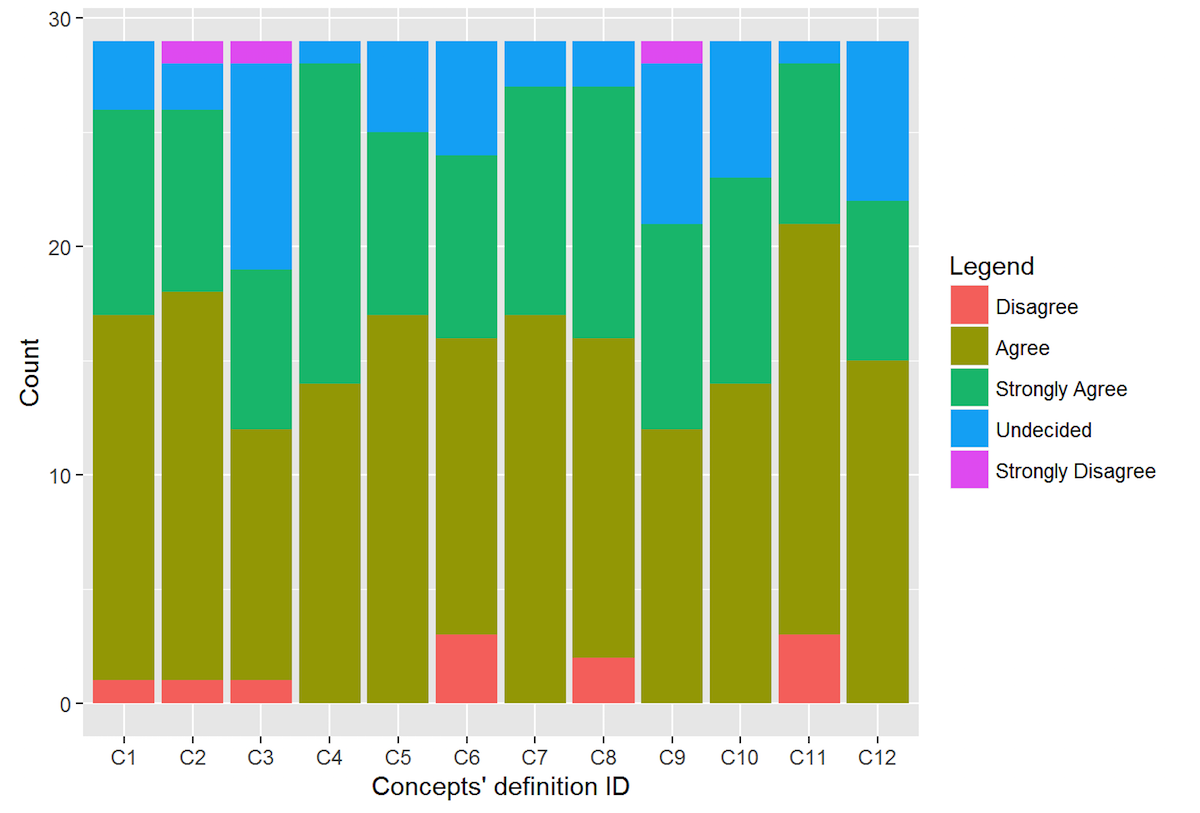
Evaluation of FBA-Ontology concepts.

Figure 3 shows how the expert of the domain assessed the 12 concept definitions provided in the first section of the survey. The majority of responses confirmed the proposed definitions. The response rate is higher for “agree” (15/29, 51.15%) and “strongly agree” (9/29, 30.75%). It is worth noticing that the concept definition 3 and 9 received the higher rate of undecided responses, respectively 9/29 (31.03%) and 7/29 (24.14%). In both cases, the items were probably ambiguous to the experts and not straightforward. The definition number 3 is: “The function of a behavior is the reason that motivates a behavioral topography.” This sentence seems to wrongly suggest that a behavioral topography depends on its’ function. However, authors wanted to get a confirmation that a function determines why a certain behavior occurred. The definition number 9 is “An observer is a person who registers qualitative and quantitative information about a behavior.” This item probably does not provide enough contextual information to responders. The definition could probably be improved by adding some information about FBA and the role of the observer in the data collection of single case research designs.

**Figure 4:**
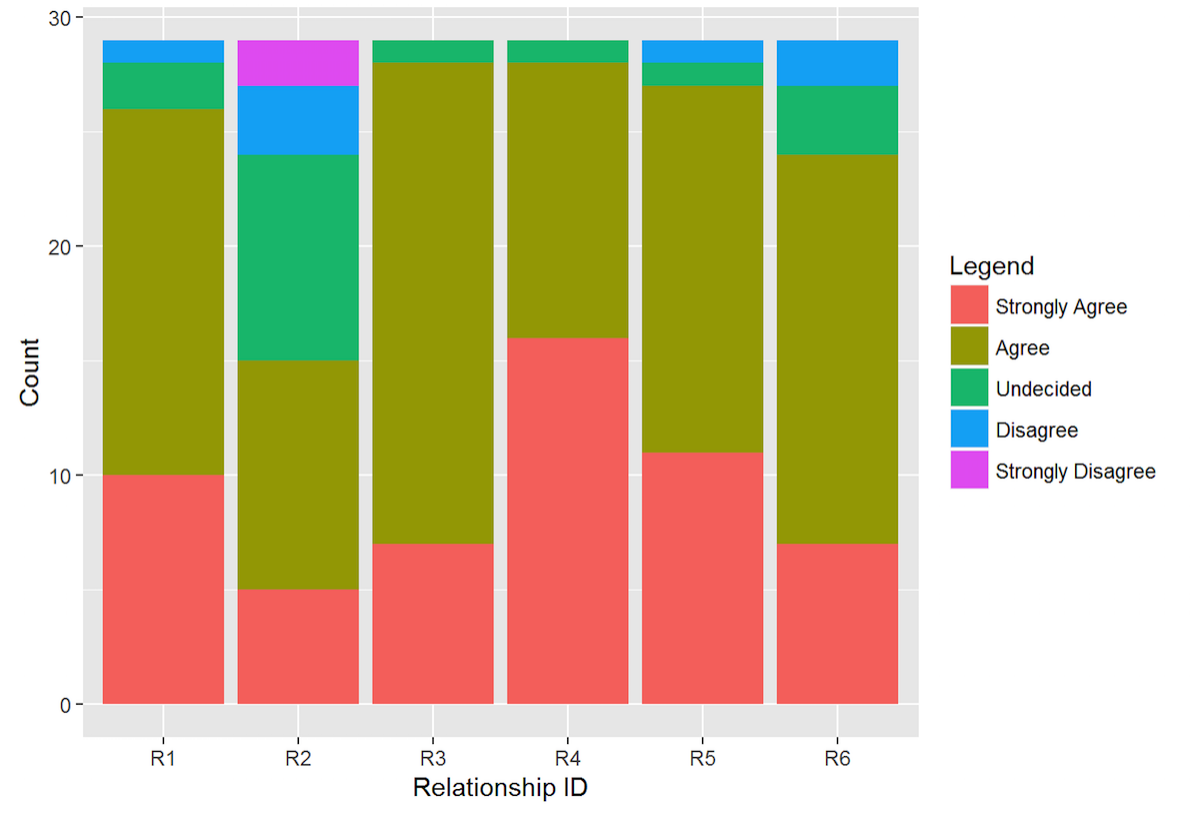
Evaluation of the relationships between FBA-Ontology concepts.

The experts’ evaluation about the correctness of the relationships between some of the most relevant concepts of the ontology is reported in Figure 4. The majority of respondents were “agree” (15/29, 52.87%) and “strongly agree” (9/29, 32.18%) with the proposed statements. The most controversial relationship is the number 2 (“A research phase must contain a minimum one measure”) that obtained the lower agreement rate of the section (18/29, 62.07%) and a large percentage of undecided (9/29, 31.03%). Once again, rather than indicating a problem with the ontology structure, the item is probably not well expressed (the verb “contain” is not self-explanatory) and lacks contextual information about the single case research design.

**Figure 5:**
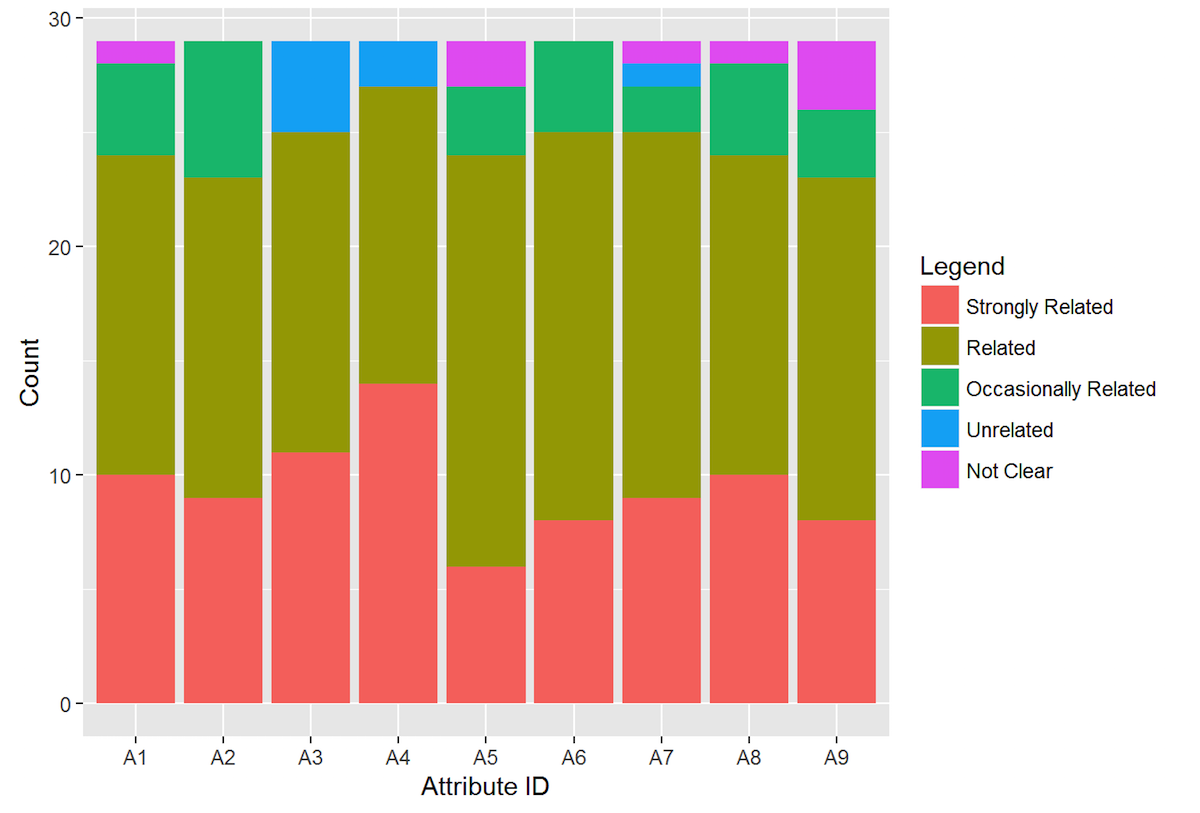
Evaluation of the FBA-Ontology attributes.

Finally, as shown in Figure 5, experts confirmed, cohesively, the relationships between the proposed concepts and their relative attributes. The higher response rate (24/29, 84.29%) was for the “related” and “strongly related” options that obtained respectively the 15/29 (51.72%) and the 9/29 (32.57%) of the overall responses. Just a few responses (4/29, 15.71%) report disagreements among the proposed attributes.

## Discussion

### Principal Findings

The FBA-Ontology describes the structure and the semantics of the FBA methods supporting the systematic collection of behavioral data, the definition of hypotheses about the function of a behavior, the consequential planning of treatment strategies, and the evidence-based evaluation of the efficacy of the applied treatments.

In the field of behavioral science, the mixing of terms and labels is frequent; this lack of common terms and shared definitions for interventions renders the aggregation of knowledge a difficult process [16]. An ontology, which provides a controlled vocabulary of agreed terms and their relationships, enables and facilitates new approaches in behavioral science. Data collected by experts are no longer collected only to be used in their research, but they can be shared, compared and integrated across experiments conducted by the whole research community. In this perspective, the FBA-Ontology represents a model able to promote the creation of new repositories, the integration, and interlinking of Linked Open Data datasets in the field of BI. It represents an open approach for sharing and exchanging data, explicating common mechanisms of action, collecting behavioral patterns, classifying contingency variables according to behavioral patterns, monitoring the statistical evidence of behavioral intervention.

Besides, the FBA-Ontology could favor the development of new applications able to support the collection of observational data in different life contexts, facilitating the interaction among practitioners and caregivers. In general, the FBA-Ontology supports the integration of several sources of data thus constituting a key element to enhance the value of the data itself. In educational settings, the presence of innovative applications could improve Lifelong Learning opportunities for teachers, parents, and clinicians to spread the use of the behavioral observation practices and the promotion of home-school relationships, to reduce the gap between research and practice. Also, the dissemination of a common communication language and the improvement of effective evidence-based decision-making processes will be advantageous from this perspective.

Concerning the ontology verification, a machine-based approach has been applied to check the logical consistency, to which the integrity of classes and properties of FBA-Ontology have not detected any violation of constraints. Moreover, the result of the questionnaire administered to domain experts confirmed that the concepts, the attributes, and the relationships between concepts defined in the FBA-Ontology are valid. These findings are particularly important for behavioral science because they contribute to improve class definitions and comparability of operational definitions, and to enable automatic and efficient meta-analysis and scientific syntheses, which, in turn, could be translated into clinical guidelines [16].

The FBA-Ontology, developed contextually to the Web Health Application for ADHD Monitoring (WHAAM) [63-65], could be a starting point to guarantee the systematic organization of behavioral knowledge and the development of future eHealth systems devoted to spreading the digital use of evidence-based assessment practices. A limitation of the work presented here concerns the lack of practical use cases in which the ontology has been adopted. This issue will be tackled in two European funded projects recently approved in the framework of the Erasmus+ program.

These projects are respectively focused on the management of social, emotional and behavioral difficulties, and the promotion of positive behaviors at school, thus offering a suitable setting to conduct further experimentations of the FBA-Ontology in a real environment. Finally, we aim to encourage not only the empirical application but also the use of computational tools and psychometric methods to provide the refinement of ontology in the future, aware that this field of study needs to be explored more in-depth.

### Conclusion

In this study, we developed the FBA Ontology to promote knowledge generation, organization, reuse, integration, and analysis of behavioral data. The FBA Ontology is composed of concepts that describe the process of gathering information about behavior to determine its function and design effective intervention plans.

The study presented the assessment of the ontology by a group of experts in the domain. Results from the human-based evaluation confirmed that the ontology concepts, attributes, and relationships between concepts are valid. Moreover, the analysis provided by automatic tools has not identified anomalies in the ontology definition. Further research involving the creation and the interlink of repositories based on the behavioral data would contribute to highlight the importance of the aggregation and sharing of information in this domain.

## Abbreviations

ADHD: Attention Deficit Hyperactivity Disorder
BI: behavioral interventions
EBP: evidence-based practice
FBA: functional behavioral assessment
WHAAM: web health application for ADHD monitoring
